# Awake intubation creates feelings of being in a vulnerable situation but cared for in safe hands: a qualitative study

**DOI:** 10.1186/s12871-016-0240-z

**Published:** 2016-08-30

**Authors:** Kati Knudsen, Ulrica Nilsson, Marieann Högman, Ulrika Pöder

**Affiliations:** 1 Department of Public Health and Caring Sciences, Uppsala University, SE 751 22 Uppsala, Sweden; 2 Department of Health and Caring Sciences, University of Gävle, Kungsbäcksvägen 47, SE 801 76 Gävle, Sweden; 3 Centre for Research & Development, Uppsala University/Region Gävleborg, SE 801 88 Gävle, Sweden; 4 Faculty of Medicine and Health, School of Health Sciences, Örebro University, SE 701 82 Örebro, Sweden; 5 Department of Medical Sciences, Uppsala University, SE 751 22 Uppsala, Sweden

**Keywords:** Awake fiberoptic intubation, Anaesthesia care, Qualitative study

## Abstract

**Background:**

Awake fiberoptic intubation is an alternative procedure for securing the airway and is a recommended option when a difficult airway is expected. The aim of the present study was to describe patient experiences with this procedure.

**Methods:**

A qualitative, descriptive design was used and patients were recruited from three county hospitals and one university hospital in Sweden. Data was collected by semi-structured interviews with 13 patients who underwent awake fiberoptic intubation. A qualitative content analysis extracted theme, categories, and subcategories.

**Results:**

From the patient statements, one main theme emerged, *feelings of being in a vulnerable situation but cared for in safe hands,* which were described in five categories with 15 subcategories. The categories were: a need for tailored information, distress and fear of the intubation, acceptance and trust of the staff’s competence, professional caring and support, and no hesitation about new awake intubation. The patients felt they lacked information about what to expect and relied on the professionals’ expertise. Some patients felt overwhelmed by the information they were given and wanted less specific information about the equipment used but more information about how they would be cared for in the operating room. Undergoing awake intubation was an acceptable experience for most patients, whereas others experienced it as being painful and terrifying because they felt they could not breathe or communicate during the procedure itself.

**Conclusions:**

Tailored information about what to expect, ensuring eye contact and breathing instruction during the procedure seems to reduce patient distress when undergoing awake fiberoptic intubation. Most of the patients would not hesitate to undergo awake intubation again in the future if needed.

## Background

Awake fiberoptic intubation via either the nasal or oral route is an alternative procedure for securing the airway and is a recommended option when a difficult airway is expected [1, 2]. Almost 500 000 general anaesthetic procedures are performed annually in Sweden [3]. Whether a patient is intubated while awake or not, they worry about being anaesthetised and intubated. For example, those who perceive stress and worry regarding the anaesthesia and surgery preoperatively report more anxiety and discomfort postoperatively [4], and patients who have experienced an awake endotracheal intubation report more feelings of discomfort, sensations of suffocation, and hoarseness than those who undergo a traditional endotracheal intubation [5]. Schnack et al. [6] studied discomfort and fear among patients who underwent awake versus anaesthetised endotracheal intubation using questionnaires from a database. Discomfort was reported more frequently following awake endotracheal intubation than conventional intubation. Bernasconi et al. [7] assessed patient satisfaction when undergoing flexible bronchoscopy for the first time and found that a majority tolerated the procedure well and would return for a repeated bronchoscopy if needed. One can speculate if patients who have undergone awake fiberoptic intubation experience similar feelings as those patients above since the procedures are nearly the same, but clinical experience and previous research contradict this hypothesis. To the best of our knowledge, no study has explored patient experiences of being awake while intubated in an anaesthesia care setting. Feedback from patients about their experiences with the procedure is useful information that may contribute to improvements in the quality of care. The present study aimed to describe patient experiences with awake fiberoptic intubation.

## Methods

A qualitative descriptive design was used [8]. The regional Ethical Review Board of Uppsala approved the study, February 2013 (D no. 2012/546/2).

Patients were recruited from three county hospitals and one university hospital in Sweden, between autumn 2013 to the spring of year 2015 (one hospital) and between autumn 2014 to the spring of year 2015 (three hospitals). The annual number of awake fiberoptic intubations at each hospital is estimated to 20 per year. Prior to anaesthesia, each patient was preoperatively airway assessed by anaesthesiologists, who also explained the procedure in accordance with their local standardized approach. All awake fiberoptic intubations were performed by an anaesthesiologist assisted by a nurse anaesthetist. A flexible fiberoptic intubation scope (Karl Storz, Tuttlingen, Germany) or flexible bronchoscope (Olympus Optical Co.Tokyo, Japan) was used. The anesthesia drugs were administrated at the beginning of the procedure before the intubation and were titrated according to the patient’s age and body weight, see Table 1. Endotracheal tube (Mallinckrodt, covidien) size 6 or 7 inner diameter was used in women and tube size 7 or 8 in men. When correct placement was confirmed general anaesthesia was induced. Anaesthesia was subsequently maintained with oxygen in nitrous oxide and sevorane or propofol with target controlled infuser.

**Table 1:** Anesthesia drugs pre and post intubation in patients undergoing awake fiberoptic intubation, *n* = 13

	n
Pre intubation:	
Lidocaine, 1 % solution spray, 3–5 spray orally	8
Lidocain, 1 % solution spray orally combination with Lidocaine 4 %, 3–4 ml i.v. in the cricothyroid membrane	3
Glycopyrrolate, 0.2–0.4 mg i.v.	6
Midazolam, 2–2.5 mg i.v.	5
Fentanyl, 0.002 mg/kg or Morphine, 0.05–2.5 mg/kg or Ketobemidom, 0.1 mg/kg i.v.	11
Post intubation:	
Propofol, 1.5–2.5 mg/kg i.v.	13
Remifentanil, 0.5–1 ug/kg/min i.v.	11
Sevofluran, inhalation	9

The inclusion criteria were patients aged ≥ 18 years who underwent awake nasal or oral fiberoptic intubation. The exclusion criteria were earlier experiences of being awake while intubated, or not speaking Swedish. A consecutive sampling procedure was used, in total 15 patients reported to undergo or had undergone an awake fiberoptic intubation during the study period. Patients who should undergo an awake intubation (*n* = 8) received written information about the study purpose and the procedure during their preoperative meeting with the anaesthesiologists. Those patients (*n* = 5) who had already undergone an awake fiberoptic intubation the written information was given 1–7 days postoperatively. Patients who agreed to be contacted by the first author (KK) were asked for verbal informed consent over the telephone 1–3 days after written information was administrated. Written informed consent was obtained on the day of the interviews. A semi-structured interview guide [8] was developed: “What is your experience with general anaesthesia in the past”? “What information was given to you before your awake intubation was performed”? “What is your experience with awake intubation”? To clarify what they meant, probing questions were asked when needed, such as, “please could you tell me more”? Or, “How did you mean”? And “How did you felt about that”? Furthermore, the patients were asked about their views of undergoing awake endotracheal intubation in the future. At the end of the interview, the researcher summarised what had been said and the patient could then add further information if needed and clarify whether anything was misinterpreted. The first author conducted two audio-recorded interviews with each patient. The first interview was conducted at the hospital or, if the patient preferred, in their own home 1–21 days (median 8) postoperatively. The interviews lasted between 19 and 61 min (median 30). A follow-up interview was conducted over the telephone approximately 4 weeks after the first interview in order to give the patient an opportunity to clarify and verify what had been said. The second interview lasted less than 30 min.

Qualitative content analysis was performed [9]. All audio recordings of the interviews were transcribed verbatim. The inductive content analysis was performed in several steps as described by Graneheim and Lundman [10]: statements and phrases (meaning units) corresponding to the aim of the study were identified, shortened to condensed meaning units, without loss of the core. The condensed meaning units were labelled with a code. The codes were compared based on similarities and differences and sorted into categories, subcategories and a theme, in dialogue with the co-authors. Examples of the analysis process are presented in Table 2. The authors met during the analysis process to discuss the upcoming subcategories, categories and themes until consensus was achieved. Verbatim quotes from the patients’ statements are presented to illustrate the findings. The authors had a pre-understanding of the phenomenon from clinical working experiences in anaesthesia (KK, MH, UN) and/or intensive care (UP, MH), which was actively reflected upon during the analysis to minimise interpretive bias [11].

**Table 2:** Examples of the analysis process, from meaning unit to theme

Meaning unit	Condensed meaning unit	Code	Subcategory	Category	Theme
I thought that the tube should be inserted when I had fallen asleep, but there must be some reason why I need to be awake	There must be some reason why I need to be awake	Unclear why awake intubation	A need for more specific information about what to expect	A need for tailored information	Feelings of being in a vulnerable situation but cared for in safe hands
If they say it is the best choice, so it will be. They know best. I was calm, he [anaesthesiologist] said it was the safest way for me.	They know best, it was the safest way for me.	The staff know best	Confidence with staff choices	Acceptance and trust in the staffs competence	

## Results

Of 15 eligible patients, two declined and 13 agreed to participate in the study. Eight of the patients were included preoperatively and five postoperatively. The variation in background characteristics, collected from the patients’ anaesthesia records, and the reasons for requiring awake fiberoptic intubation are described in Table 3.

**Table 3:** Descriptive characteristics of patient data and reasons for requiring awake fiberoptic intubation

Patients	*n* = 13
Mean age years (min – max)	53 (28–77)
Male/female	7/6
Mean height cm (min-max)	166 (146–177)
Mean weight kg (min-max)	72 (44–105)
Mean BMI (min-max)	27 (19–38)
ASA I/II/III/IV	2/4/2/5
Mallampati class I/II/III/IV	6/2/2/3
Orally/nasally awake fiberoptic intubation	6/7
Reasons for awake fiberoptic intubation	
o Cervical spine disease, i.e. rheumatoid arthritis	5
o Head and neck abnormality, i.e. supraglottic cancer, oral abscess	2
o Thyroidea abnormality	3
o Upper airway trauma, i.e. acute pharyngeal bleeding, mandible fracture	3
Previous experience with anaesthesia, yes/no	10/3

From the patient statements, one main theme, feeling of being in a vulnerable situation but cared for in safe hands, emerged that are described in five categories with 15 subcategories (Table 4). Subcategories are written in italics within the text illustrating each category.

**Table 4:** Descriptions of the theme *Feelings of being in a vulnerable situation but cared for in safe hands* into categories and subcategories

Category	Subcategories
A need for tailored information	■ A wish for general information about the procedure, without technical details of the equipment. ■ A need for more specific information about what to expect
Distress and fear of the intubation	■ Fear of throwing up ■ Fear of the local anaesthetic and the tube size ■ Feelings of discomfort, coughing, suffocation, and pain
Acceptance and trust of the staff’s competence	■ Limited knowledge about anaesthesia ■ Confidence with staff choices
Professional caring and support	■ Comfort on the operating table ■ Confidence in the staff ■ Vulnerable situation, but treated in a careful manner ■ A wish for eye contact ■ A wish for breathing instruction
No hesitation about new awake intubation	■ Acceptable experience, and there are worse things ■ It went rapidly ■ It is the same phenomenon as gastroscopy ■ Forget unpleasant memories

### A need for tailored information

Patients described having different concerns about how they wished to be involved and how much information they needed before the intubation. Some *wished for general information about the procedure, without technical details of the equipment*. Thus they were not in the emotional state to hear or read about it. The detailed information was considered to make it scarier than to be supportive. In contrast, some patients described *a need for more specific information about what to expect*, as they were not clear on why they should undergo awake intubation. They would have appreciated more information about the procedure and how they would experience the awake intubation. Not knowing what to expect or having reduced or no control over what would happen in relation to the intubation provoked a sense of being in a vulnerable situation. It was important to patients that the anaesthetists prepared them carefully and gave them tailored information, as it helped them cope and to feel confident both before and during the procedure. Some patients wanted illustrative information to read at home.

*“If they become too detailed, it might be that I get more upset… if they start talking about the tubing thing that should be put down your throat and that might be a little problematic and they are going to anaesthetise after they have the tube in place and so on… at that point it was a little bit scary, because I know how it is when I got something in my throat, but I do not know if they can do it another way”* (Male No 8).

### Distress and fear of the intubation

Patients described knowing that they would undergo awake intubation as emotionally stressful. Interpretations of the information received about the procedure resulted in fear of throwing up during the procedure due to potential provocation of the gag reflex by the tube, fear of the local anaesthetics, and fears about the size of the tube. Some of the patients’ fears became reality during the procedure with the local anaesthetic solution sprayed into the throat and the injection into the cricothyroid membrane evoking feelings of discomfort, coughing, and suffocation. The patients stated that this was the most unpleasant experience during the whole procedure. The intubation was extremely painful for some, who felt when coughing during the procedure that the nose bone would explode as the tube was inserted. On the other hand, patients who previously experienced upper intestinal gastroscopy procedures described awake intubation as nearly the same phenomenon and that there was nothing to fear.

*“It sounded so nasty and I don’t really know what the instruments look like, I thought, oh God how will it be done, I imagine some big instrument that would be put down my throat”* (Female No 4).

*“It felt like I could not swallow, like I would suffocate on the fluid, like as I cough and throw up at the same time and then it burns”* (Female No 5).

### Acceptance and trust of the staff’s competence

Trust in the anaesthetists’ competence was a common description. Patients accepted the anaesthesiologist’s choice of anaesthetic method because they had *limited knowledge about anaesthesia* and they lacked knowledge about other alternatives of how to being anesthetised. The patients understood that being awake during the procedures was for their own security and felt *confidence with the staff choices*. They expected that the anaesthesiologist knew best, and many stated that they did not consider lack of influence a problem. Some patients prepared for the procedure by relaxing and meditating, which helped them keep calm.

*“It was just to realise that I must leave myself in other people’s hands. I had full confidence, I meditated and calmed down… There is nothing I can do now, so I have to trust you and your colleagues, those who are going to do this, I must resign myself to it”* (Male No 8).

### Professional caring and support

Patients expressed worries about their *comfort on the operating table*, whether they would be positioned so their head had enough support or if they would be strapped down and unable to move. However, when they arrived at the operating room the anaesthetists had prepared the operating table with a comfortable mattress, a pillow to support their head, and a warm blanket to cover them. This made them feel less exposed and more confident. Even if the patients stated that they had full *confidence in the staff* and felt that they were *treated in a careful manner,* some of them at the same time felt vulnerable due to the environment in the operating room and due to the hospital clothes.

*“It’s uncomfortable to get something in your throat, but it went well when I got sedated so you feel calm, but it was still unpleasant… and then you feel exposed… because the clothes you need to wear … but otherwise it went well …”*(Female No 9).

Professional support from the anaesthetists was described as important and helped the patients cope during the procedure. The patients stated that the staff were friendly and acted professionally. Patients described feeling calm when the anaesthetists talked with them continuously during the procedure and felt they were treated in a careful manner when their individual needs were met. When they felt scared to be awake during the intubation, the anaesthesiologist told them what was happening as the nurse anaesthetist supported and held their hands. Patients described feeling more involved and confident during the procedure when the anaesthetists were encouraging and made *eye contact* with them. It was important for the patients that the anaesthetists were in front of them and saw how they reacted. This made them feel safe and secure, even if they could not verbally respond. However, patients stated that better support could have been offered if the anaesthetists had *instructed* them on how and when they should *breathe* during the procedure. Deep breathing during tube insertion and pausing the insertion when breathing out was perceived to decrease the pain.

*“Someone should have been in front of me… and established eye contact with me… It’s not so easy to say something to someone that you do not see…To do this together feel that you are involved…If I had had eye contact I would have felt I was more involved”* (Female No 12).

### No hesitation about new awake intubation

Most patients described undergoing awake intubation as an *acceptable experience and that there are worse things*. They described feeling the tube going down, but *it went rapidly*. Patients also fell asleep as soon as the tube was in place. Most of the patients stated that they would not hesitate to undergo awake intubation again in the future if needed. Those patients with previously experience of gastroscopy stated that it is nearly the same procedure.

*“It’s just when they passed it through, you feel it when it goes down, but it doesn’t hurt or something like that, not for me anyway…I have only the memory of someone spraying in the throat to remove the gag reflex, just as if you go on a “gastro” it’s the same phenomenon”* (Male No 1).

Patients described *forgetting the unpleasant memories* afterwards. One female drew a parallel between the awake intubation and childbirth; you forget the really distressing moment after the successful procedure.

*“It is like childbirth; you forget how nasty and painful it was, but should they say that you need to do it again, I would probably do it, if it was for my security, and you forget afterward when you are feeling healthy again”* (Female No 5).

A few patients had no memories at all; an older patient who underwent awake intubation stated that the worst thing about the whole procedure was that he did not remember anything from the time he went to the hospital until he was home again, concluding that 5 days of his life was gone. He could not understand why he did not remember anything and that felt strange.

## Discussion

The main findings of this study showed that undergoing awake intubation was an acceptable experience for most patients, whereas others experienced it as being painful and terrifying. The application of local anaesthetic evoked feelings of discomfort, coughing, and suffocation. The patients also felt they lacked information about what to expect and relied on the professionals’ expertise. However, some patients felt overwhelmed by the information they were given and wanted less specific information about the equipment used but more information about how they would be cared for in the operating room.

Knowing what to expect increased the patients’ feelings of control and ability to cope during the procedure and they found it easier to undergo awake intubation if the anaesthetists supported and instructed them on how to breathe during the procedure. These findings are in line with earlier studies regarding pre-operative information [12–14].

The majority of patients had an acceptable experience with the awake intubation, despite the emotional stress. Schnack et al. [6] reported that awake intubation was more discomforting than conventional intubation and patients feared the operation more than the anaesthesia and intubation itself. However, no specific discomfort was reported in their study following the use of local anaesthetic. In the present study, several patients felt that the worst part of the whole procedure was discomfort following application of the local anaesthetic, including pain, coughing, and feelings of suffocation. In general, the nasal awake intubation procedure has been described as more comfortable compared to the oral procedure, due to the lower risk of stimulating the gag reflex [2]. However, this difference was not shown in our study, possible due to the small sample size. If the patients feared the surgery more than the anaesthesia procedure or vice versa was not illuminated in the present study. One possible explanation for discomfort following awake intubation could be that the local anaesthetic was poorly applied, or that the administration of sedative to keep the patient calm was not enough, or that the injection of glycopyrronium to reduces airway secretions was lacking [15], or the tube size was not optimal [16]. Yet, if a different patient characteristic has an impact on patients’ experience of discomfort during an awake intubation has to be explored further. It is a dilemma when patients need to be sedated enough to make the procedure more tolerable and to alleviate unpleasant experience, but on the other hand, they need to be able to follow instructions and commands during the procedure. Patients are vulnerable during an awake intubation because it is impossible to vocally communicate when the tube is inserted. Patients need to trust the staff caring for them. In our study, patients expressed the importance of eye contact during the procedure, so they could communicate with staff if something felt wrong during the procedure. Similar findings are reported by Karlsson et al. [17] but during regional anaesthesia. Even if their study did not focus of being awake while the intubation *per se*, the patients expressed communication difficulties with the staff. By having eye contact and give breathing instructions may reduce the psychological distress and thereby help patients to feel more comfortable in the situation.

It is important to take patient experiences into account to improve the quality of care*.* One example is to give patients tailored information on what to expect, including a description of how awake intubation could be experienced. Different formats could be used, such as a short informative brochure about how the patient will be cared for in the operating room, which may increase their feeling of control. We also suggest the information contain breathing instructions for tube insertion to make the procedure easier and more comfortable. Previous studies reported that patients who receive written information preoperatively feel more involved and are more satisfied with their care than those who do not [18, 19].

Most patients had vague memories of the procedure, possibly due to the sedation. A lack of memories after anaesthesia procedures is well known [20]. In the present study, most patients experienced feeling the tube going down and woke up with the same feelings as when they were falling asleep, but others felt confused. However, disorientation after anaesthetics is not uncommon, especially among older patients, who are more predisposed to postoperative disorientation than younger patients [21]. Despite experiencing some discomfort, patients stated that awake intubation was an acceptable experience and they would not hesitate to undergo endotracheal intubation again if needed in the future. A similar finding was reported previously [7] for patients undergoing flexible bronchoscopy, which is nearly the same procedure in clinical practice.

Strength of the present study is that patients of both sexes and varying ages and experiences were included. The sample size was small in this study. However, data saturation was achieved after nine of the 13 interviews. Hence, referring to the method, further interviews could probably not bring new information that could change the outcome [9]. To strengthen the credibility and dependability of the findings, a detailed description of the analysis is provided and with verbatim quotations from the patient interviews. The anaesthetists were informed about the study, which could have had an effect on the anaesthesia procedure, thereby also patients’ experience of being awake intubated, which can be a limitation. The small sample size and qualitative study design implies caution to widely generalize the findings, and that the transferability of the findings to other settings at the end is for the reader to decide [9]. The findings provide useful information for clinicians and may contribute to improvements in the quality of care.

## Conclusions

Professional support from the anaesthetists helped patients cope during the procedure. This includes breathing instructions in connection to the tube insertion and to ensure eye contact for helping patients to communicate with the staff. If patients receive tailored information about what to expect and how they could experience the procedure, it could reduce their distress. However, the majority of patients found awake intubation to be quite acceptable and would not hesitate to undergo such intubation again in the future if needed.
